# Managing accidental left pulmonary vein transection during segmentectomy

**DOI:** 10.1016/j.xjtc.2025.06.021

**Published:** 2025-07-01

**Authors:** Kohei Hashimoto, Yoshifumi Hirata, Kazuharu Suda, Keisei Tachibana, Ryota Tanaka, Haruhiko Kondo

**Affiliations:** Division of Thoracic Surgery, Kyorin University, Tokyo, Japan

**Figure:**
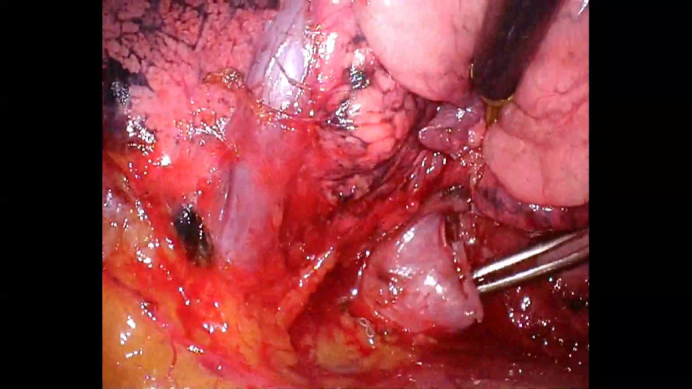
Accidentally transected left basal vein.

**Central Message**

A routine check of both pulmonary veins is key to accurately identifying venous anatomy during left pulmonary resections. Even in the era of segmentectomy for lung cancer, this step remains important.

After 2 large randomized trials^1^^,^^2^ demonstrating the oncologic noninferiority of segmentectomy compared with lobectomy for small, peripheral lung cancers, segmentectomy has become a standard surgical option. Its clinical application has rapidly expanded in recent years. However, during left-sided pulmonary resections, particular caution is warranted when identifying pulmonary venous anatomy—especially in minimally invasive approaches, where the working space is more limited than in open thoracotomy. We present a case of successful repair of an accidentally transected left basal vein during lingular segmentectomy, performed in a patient with previous right upper lobectomy, highlighting both the technical considerations and the importance of careful venous identification.

## Clinical Summary

**Figure 1:**
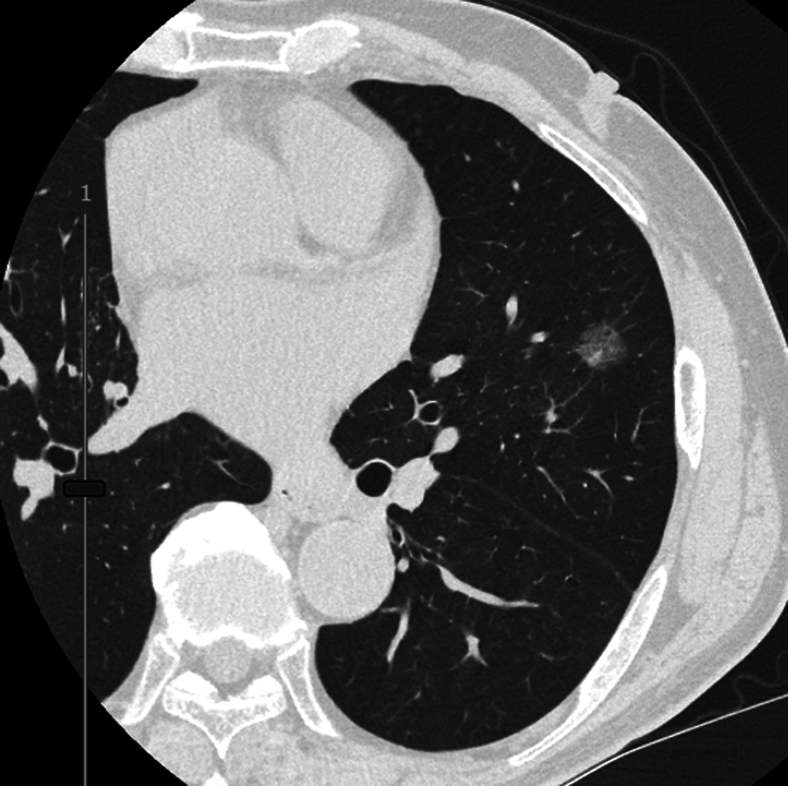
A computed tomography showing the target lung cancer in the left lingula.

A 70-year-old man presented with a part-solid ground-glass opacity in the lingular segment (Figure 1). He had previously undergone right upper lobectomy for stage IA lung adenocarcinoma 18 months earlier. During routine follow-up, a gradually enlarging ground-glass opacity was detected in the lingula. Medical history included history of smoking, atrial fibrillation, and hyperlipidemia. Forced expiratory volume in 1 second was 2.2 L (80% of the predicted value). However, the % forced expiratory volume in 1 second was 65%, consistent with mild obstructive impairment.

**Figure 2:**
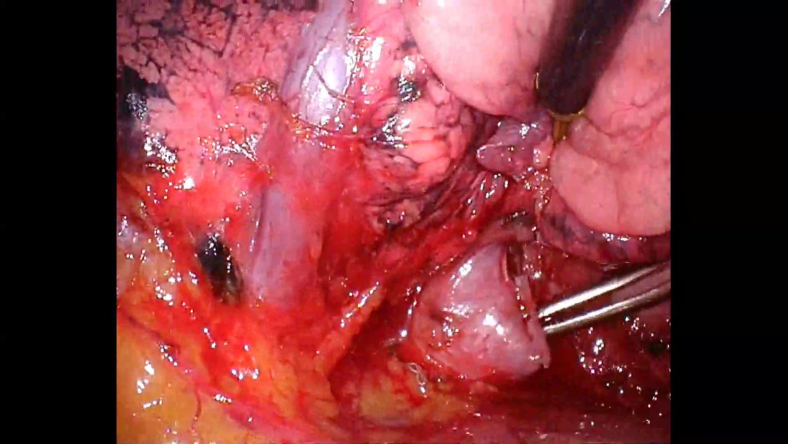
Accidentally transected left basal vein.

During segmentectomy performed by a junior surgeon, a pulmonary vein thought to be draining the upper lobe was dissected and the lower half branch was divided using a vascular stapler. The vein was located below the interlobar fissure and was assumed to be V4+5. However, further dissection revealed that the previously divided vessel was not the lingular vein but the basal vein (Figure 2, Video 1). At this point, a senior surgeon was called into the operating room. The critical question was whether to attempt vascular reconstruction or proceed with left basal segmentectomy. Because the patient had undergone a right upper lobectomy, left basal and ligular sementectomies were thought to be potentially hazardous.

We proceeded with thoracotomy and obtained vascular control of the left main pulmonary artery and the superior pulmonary vein. The divided pulmonary vein stump was carefully dissected toward the lung. Heparin (2000 units) was administered systemically. The left main pulmonary artery was clamped, the superior pulmonary vein was snared to control backflow, and the inferior pulmonary vein near the left atrium was clamped. The V6 was also snared to control the flow of bronchial artery into the anastomosis site. Staple lines at both venous stumps were carefully removed. The vein was reconstructed using 5-0 polypropylene suture in a running fashion, with great care taken to avoid obstructing adjacent branches. After deairing, vascular clamps were removed. A lingular segmentectomy was then completed. Intraoperatively, the left lower lobe showed adequate perfusion and inflation (Video 2).

Operation time was 3 hours and 39 minutes, including a pulmonary clamp time of 28 minutes. Blood loss was 245 mL. Postoperative recovery was smooth. Anticoagulant was not used. Pathologically, this was a lepidic adenocarcinoma (p-stage IA1) with negative resection margins. Follow-up enhanced computed tomography at 6 months showed that left lower lobe remained well-expanded, with no evidence of stenosis or kinking at the anastomotic site. Peripheral venous branches distal to the anastomosis also showed preserved flow and expansion (Video 3). Up to the 1-year follow-up, no parenchymal changes were noted in the left lower lung on the computed tomography scan. Written informed consent for publication was obtained from the patient; institutional review board approval was not required.

## Comments

We present a successful reconstruction of the left basal pulmonary vein after its accidental transection during a planned lingular segmentectomy. Although there are a few reports describing reapproximation of an inadvertently transected left inferior pulmonary vein to the left atrium during planned left upper lobectomy,^3^ to our knowledge, no previous reports have described reconstruction of a transected basal pulmonary vein during segmentectomy.

Although such repair may not always be feasible—particularly when the transection occurs across multiple peripheral branches—our case demonstrates that segmental pulmonary vein reconstruction is technically possible under appropriate conditions. This experience underscores the importance of meticulous identification of both the superior and inferior pulmonary veins to avoid such complications. This remains paramount in the coming era of segmentectomy. It is also advisable to dissect the vein, artery, and fissure as much as possible before transecting any vessels during segmentectomy. Furthermore, surgeons should maintain a low threshold for seeking assistance from senior staff until they have gained sufficient experience with the procedure.

## Conclusions

To prevent misidentification of venous anatomy, it is important to routinely check both pulmonary veins during left lung resection. Every effort should be made to prevent accidental transection, including meticulous dissection of anatomical structures before division and actively seeking assistance from senior staff during the learning phase of segmentectomy. If the proper steps are taken, accidentally transected pulmonary veins can be repaired.

## Conflict of Interest Statement

The authors reported no conflicts of interest.

The *Journal* policy requires editors and reviewers to disclose conflicts of interest and to decline handling or reviewing manuscripts for which they may have a conflict of interest. The editors and reviewers of this article have no conflicts of interest.

## Supplementary Data

Video 1 Misidentification of the left pulmonary veins and transection of the left pulmonary basal vein. Video available at: https://www.jtcvs.org/article/S2666-2507(25)00270-6/fulltext.

Video 2 Technical aspects of repairing an accidentally transected left pulmonary basal vein during a planned thoracoscopic left lingulectomy. Video available at: https://www.jtcvs.org/article/S2666-2507(25)00270-6/fulltext.

Video 3 Follow-up computed tomography at postoperative 6 months showed patent anastomosed vein. Video available at: https://www.jtcvs.org/article/S2666-2507(25)00270-6/fulltext.
