# Supplementary figures and images for: Managing accidental left pulmonary vein transection during segmentectomy
Source: JTCVS Tech. 2025 Jul 1;33:244–6. doi: 10.1016/j.xjtc.2025.06.021 (PMC12529727; doi:10.1016/j.xjtc.2025.06.021)

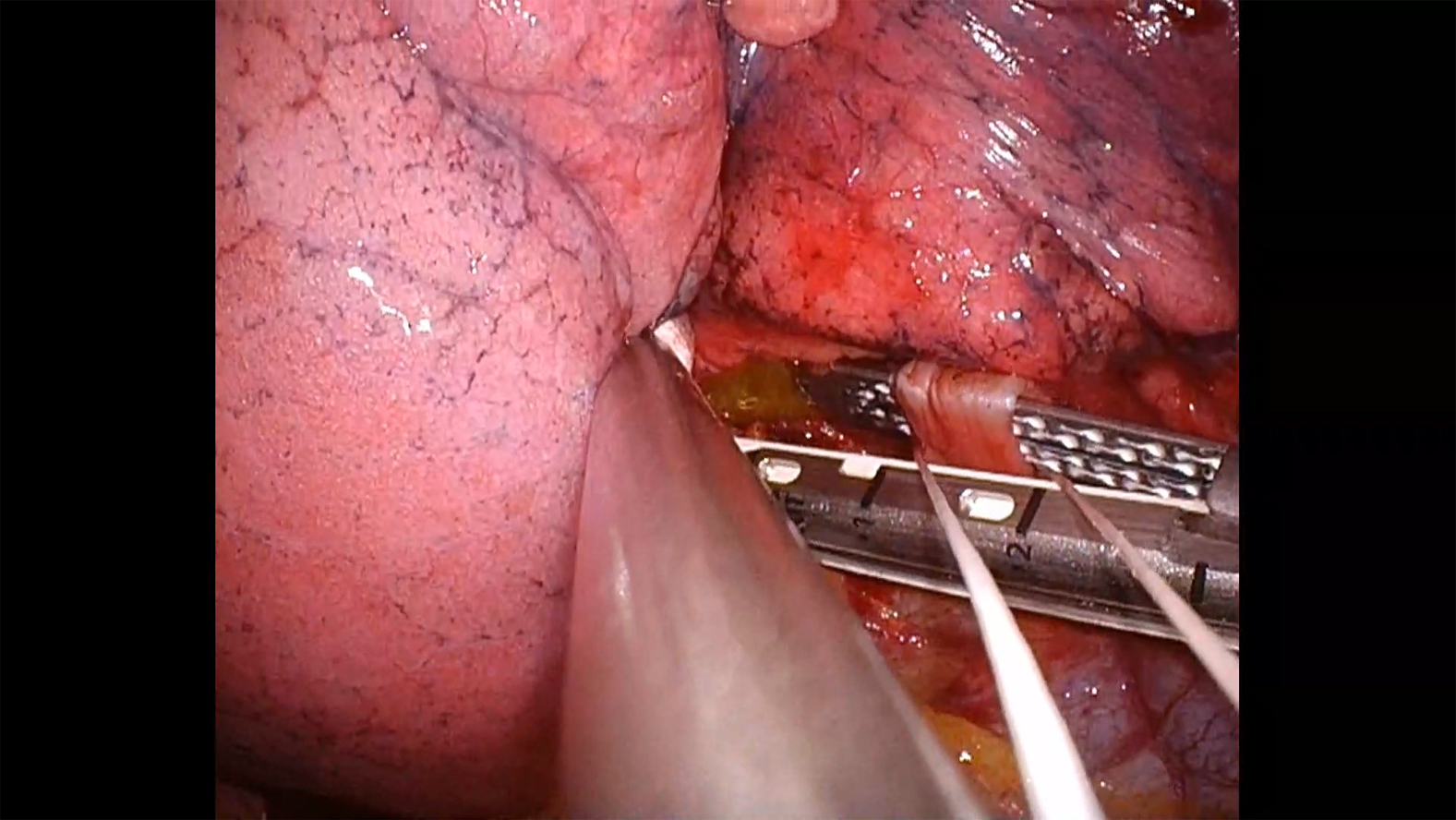

Supplement: Video 1 — Misidentification of the left pulmonary veins and transection of the left pulmonary basal vein. Video available at: https://www.jtcvs.org/article/S2666-2507(25)00270-6/fulltext. [file fx2.jpg]

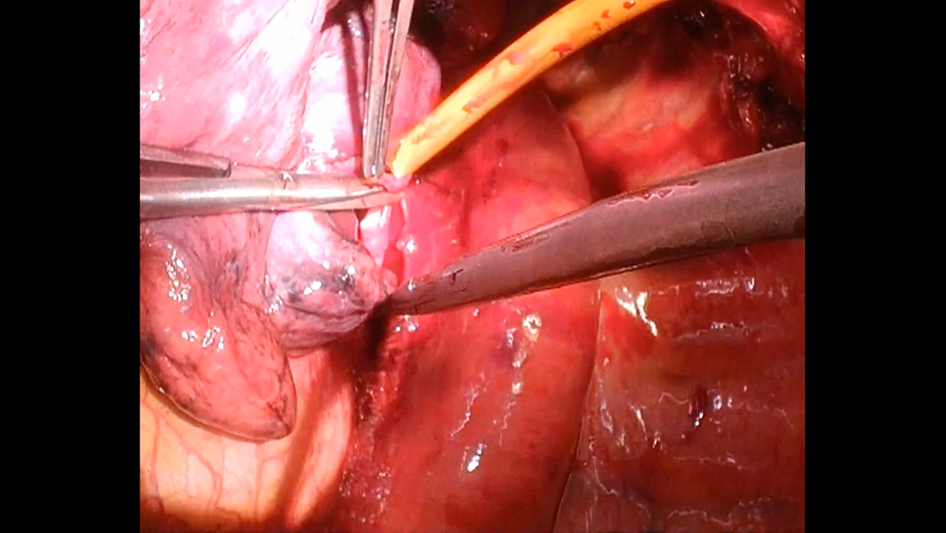

Supplement: Video 2 — Technical aspects of repairing an accidentally transected left pulmonary basal vein during a planned thoracoscopic left lingulectomy. Video available at: https://www.jtcvs.org/article/S2666-2507(25)00270-6/fulltext. [file fx3.jpg]

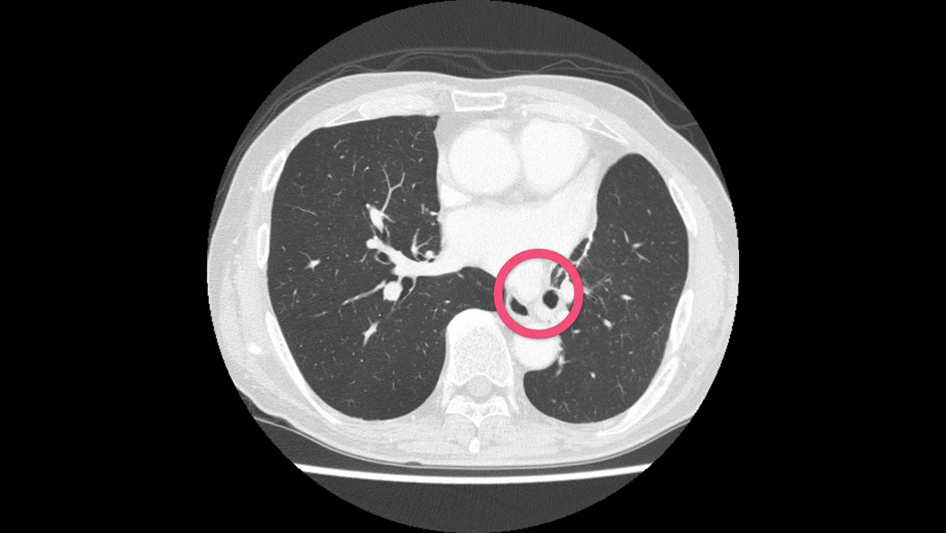

Supplement: Video 3 — Follow-up computed tomography at postoperative 6 months showed patent anastomosed vein. Video available at: https://www.jtcvs.org/article/S2666-2507(25)00270-6/fulltext. [file fx4.jpg]
